# Lung adenocarcinoma discovered during the follow-up of lung-dominant connective tissue disease: a case report and literature review

**DOI:** 10.1186/s12890-024-02975-1

**Published:** 2024-04-12

**Authors:** Zi Heng Zhu, Yi Guo, Xiao Yin Wang, Xian Wen Sun

**Affiliations:** 1 Department of Respiratory Medicine and Critical Care Medicine, Ruijin Hospital, Shanghai Jiao Tong University School of Medicine, Shanghai, China; 2 Department of Respiratory Medicine, Qingyang Hospital of Traditional Chinese Medicine, 745000 Qingyang, Gansu China

**Keywords:** Lung-dominant, Interstitial lung disease, Lung cancer, Case report, Literature review

## Abstract

Interstitial lung disease (ILD) can lead to lung cancer, which brings great challenges to differential diagnosis and comprehensive treatment. However, the clinical features of lung-dominant connective tissue disease (LD-CTD) related ILD combined with lung cancer has not been validated. We report the case of an 80-year-old woman with LD-CTD treated regularly with nintedanib who presented progressive dyspnoea and hypoxemia after recurrent viral infections. Her chest computed tomography (CT) showed aggravated interstitial fibrosis in both lower lungs with moderate right pleural effusion. Clinicians should be alert to lung cancer in patients who are experiencing poor responsiveness to treatment or acute progression of ILD. The available literatures about the differential diagnosis of clinical manifestations, imaging, treatment and prognosis of LD-CTD are reviewed and discussed in this study.

## Introduction

Interstitial lung disease (ILD) is a group of heterogeneous diffuse pulmonary diseases characterized by inflammation of the pulmonary parenchyma and alveoli and interstitial fibrosis [1]. Interstitial pneumonia is the prevalent manifestation of lung involvement in patients with lung-dominant connective tissue disease (LD-CTD). No difference was found in patients with definite connective tissue disease(CTD) and LD-CTD in terms of clinical or serological characteristics [2]. There is substantial variability across CTD in both the prevalence and pattern of ILD subtypes, and the prevalence of CTD in patients with ILD is 6-72% [3]. It has proved that ILD is a leading cause of mortality and an important prognostic factor in patients with CTD [4]. Both ILD and CTD are significant risk factors for lung cancer (LC). The morbidity of LC was approximately 10.2%∼13.0% in ILD patients, which increased to 26.6% during the 10-year follow-up period. Patients with ILD-LC experience greater all-cause mortality than do those with ILD alone (hazard ratio (HR) = 1.51, [95% confidence interval (CI) = 1.22–1.86]) [5, 6]. The risk of LC may increase during the disease course of CTD, and 5.5%∼9.0% of patients with CTD-ILD suffer from LC [7, 8]. The median overall survival time after developing LC was 7.0 months (95% CI 4.9–9.1 months), and the most common cause of death was LC [8]. Therefore, early tumour screening and regular follow-up are necessary for ILD patients. In this report, we present the case of a patient with LD-CTD following infection who was ultimately diagnosed with LC. At the same time, the imaging characteristics of LD-CTD needs to be differentiated, and virus infection was associated with an increased risk of lung cancer and worsened lung tumour progression. Additionally, we provide relevant literature to discuss the differential diagnosis of these diseases.

## Case report

**Fig. 1:**
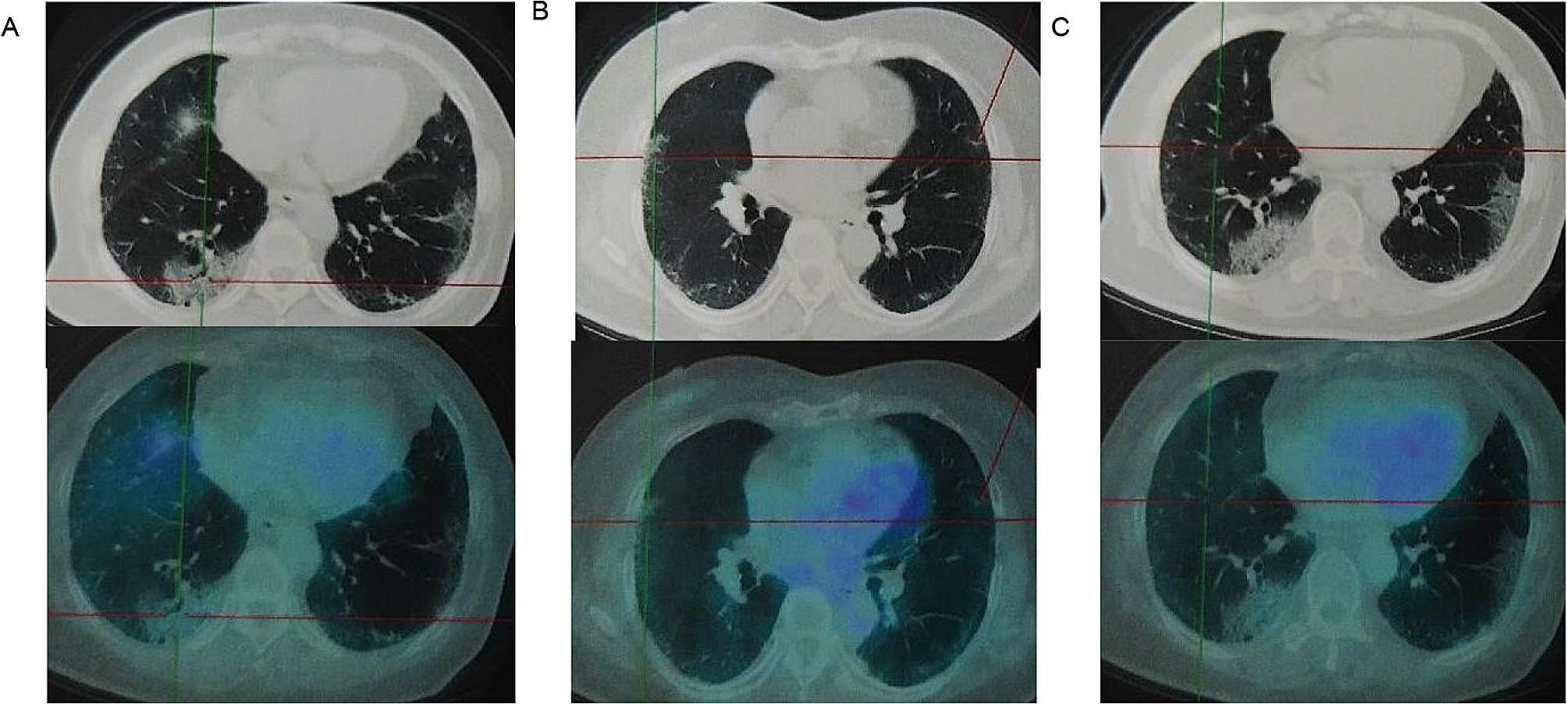
PET-CT revealed extensive interstitial changes and multiple patchy areas in both lungs with slightly increased fluorodeoxyglucose (FDG) metabolism (SUV = 1.95), and the larger lesions were in the inner and posterior basal segments of the right lower lobe

**Fig. 2:**
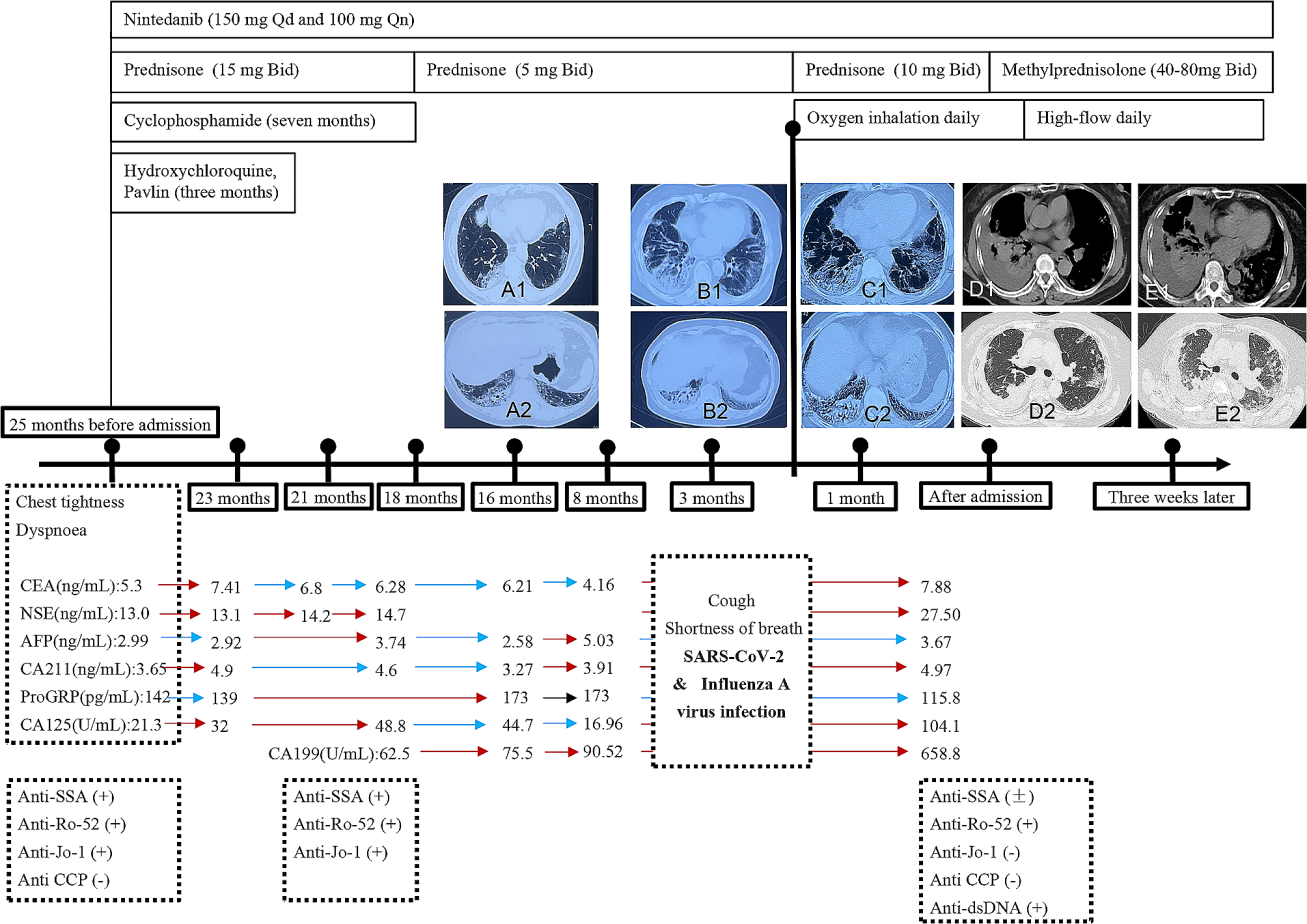
Dynamic changes of imaging, tumor markers and treatment duration. Sixteen months ago, a new small paraspinal nodule in the right lower lung was observed, and there was increased consolidation compared to that in previous scans (**A1, A2**). Three months ago, bilateral interstitial changes with increased exudation, thickened bronchial walls, and a small amount of pleural effusion on the right side were observed (**B1, B2**). One month prior, there was significant progression of right pleural effusion (**C1, C2**). Chest CT after admission showing bilateral pulmonary interstitial lesions with moderate pleural effusion on the right side and atelectasis in the right lower lobe (**D1, D2**). Three weeks after admission (before thoracic puncture and drainage), the extent of major interstitial lesions in both lungs increased, and there was a significant increase in pleural fluid on the right side (**E1, E2**)

An 80-year-old woman who had chest tightness and dyspnea that worsened after physical exertion without cough, expectoration, or reduced activity tolerance twenty-five months prior. Chest CT showed bilateral interstitial pneumonia. The patient tested positive for Anti-Sjogren’s syndrome A (SSA), anti-Ro-52 and anti-Jo-1 antibodies. The serum level of carcinoembryonic antigen (CEA) was 5.3 ng/mL, and positron emission tomography-computed tomography (PET-CT) was used to evaluate her lungs. Extensive interstitial changes and multiple patchy areas in both lungs with slightly increased fluorodeoxyglucose (FDG) metabolism were observed (standard uptake value (SUV) = 1.95) (Fig. 1). Oral treatment consisting of nintedanib (150 mg Qd and 100 mg Qn), prednisone (15 mg Bid), hydroxychloroquine, Pavlin (for three months) and cyclophosphamide (for seven months) was initiated. Sixteen months ago, with regular follow-up, her serum level of CEA was consistently above normal, and chest CT showed a new small paraspinal nodule in the right lower lung along with increased consolidation compared to previous scans (Fig. 2. A1, A2). Sputum smears were negative for tumour cells and lacked bacteria, fungi and tuberculosis. Due to the risk of pneumothorax, the desired relief of symptoms and the potentially greater risks associated with older age, she refused bronchoscopy and lung biopsy. Moreover, her serum tumour marker levels were above the upper limit but remained stable (Fig. 2). Three months ago, the patient developed cough and shortness of breath continuously following severe acute respiratory syndrome coronavirus 2 (SARS-CoV-2) and influenza A virus infection, resulting in limited daily activities. Chest CT revealed bilateral interstitial changes with increased exudation, thickened bronchial walls, and a small amount of pleural effusion on the right side (Fig. 2. B1, B2). One month prior, with significant exacerbation of breathing difficulties, her chest CT showed significant progression of right pleural effusion compared to her previous CT (Fig. 2. C1, C2). The patient then relied on oxygen inhalation for daily activities. However, her oxygen saturation decreased to as low as 70-80% within five minutes without supplemental oxygen support. Consequently, she had difficulties tolerating even mild physical exertion. The chronic obstructive pulmonary disease (COPD) Assessment Test (CAT) score was recorded as 28, and the modified Medical Research Council (mMRC) score was 3.

The patient complained of joint pain and was clinically diagnosed RA without regular follow-up sixty years ago. She did not undergo any biopsy because of the stable disease and limited condition. A right nephrectomy was performed fifty years ago due to renal tuberculosis, and renal function was normal. She had a duodenal ulcer thirty years prior and had diabetes and hyperuricaemia two years prior. Currently, she is taking metformin (500 mg Bid) and febuxostat (20 mg Qd) orally. She denied any history of smoking, drinking, exposure to asbestos, dust, wet environment or similar hazards.

**Fig. 3:**
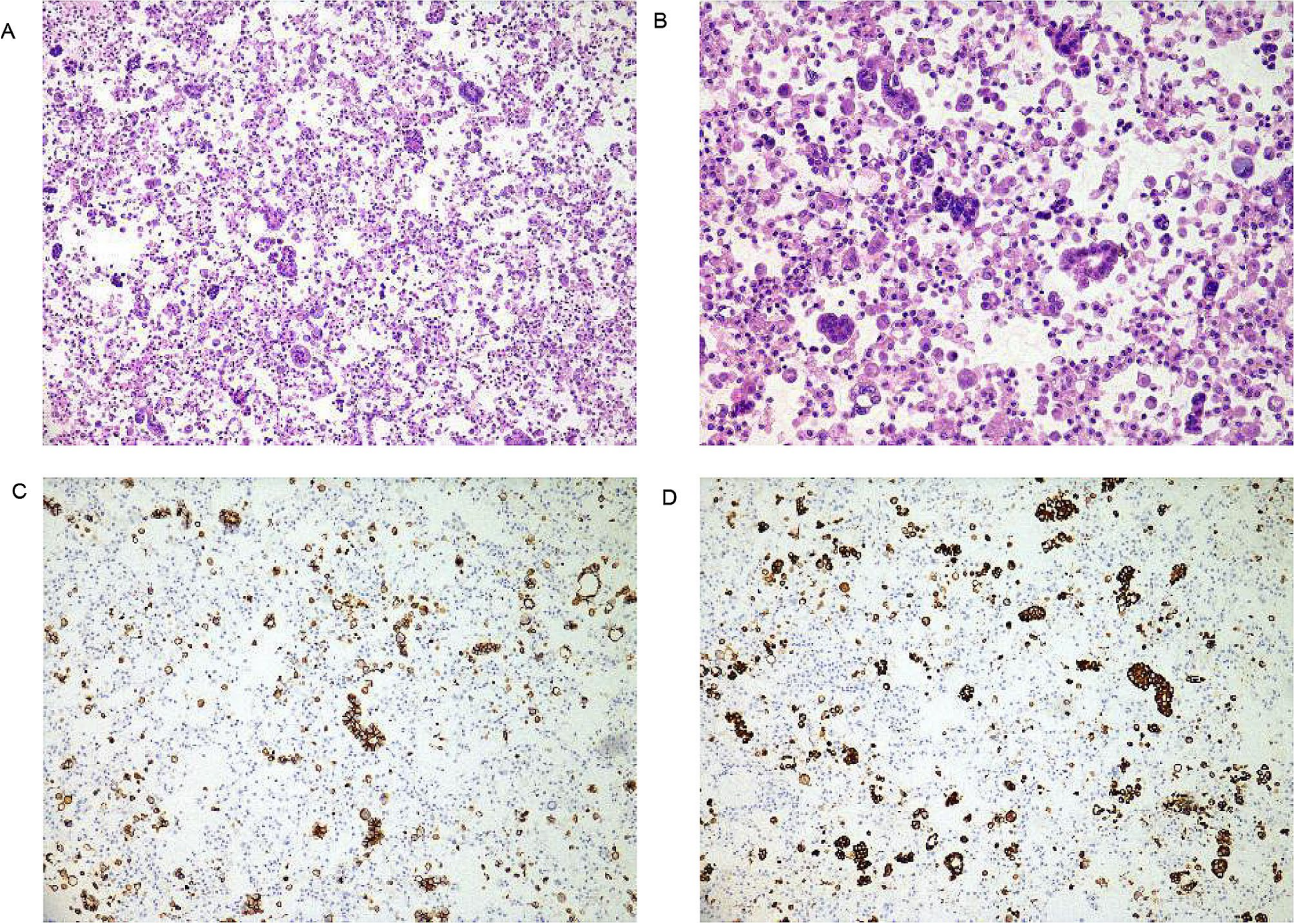
Histopathological evaluation Haematoxylin-eosin staining at a magnification of ×100 (**A**). Haematoxylin-eosin staining at a magnification of ×200 (**B**). Lymphocytes, mesothelial cells, and histiocytes were observed with very few morphologically atypical cells. E-cadherin-positive cells (**C**). MUC5AC-positive cells (**D**)

Her capillary oxygen saturation level reached 99% with nasal catheter oxygen inhalation (3 L/min). She had reduced breath sounds in the right lung and audible Velcro rales at the end of inspiration in both lower lungs. However, no clubbed digits or joint deformities were observed. The results of routine blood analysis, C-reactive protein (CRP) level, procalcitonin level and erythrocyte sedimentation rate (ESR) were within the normal range. Blood gas analysis (BAG) with oxygen inhalation at a rate of 3 L/min showed a PaO_2_ of 167 mmHg, a carbon dioxide partial pressure (PaCO_2_) of 50 mmHg, and a blood oxygen saturation (SaO_2_) of 98.5%. The serum concentration of Krebs von den Lungen-6 (KL-6) was 727.6 IU/mL. The patient tested positive for Anti-SSA and anti-RO-52 antibodies (Table 1). The chest CT showed bilateral pulmonary interstitial lesions with moderate pleural effusion on the right side and atelectasis of the right lower lobe (Fig. 2. D1, D2). Considering the increased interstitial changes in both lower lungs, we continued nintedanib, methylprednisolone sodium succinate (40 mg-80 mg Bid), and preventive anticoagulation. The next-generation sequencing (NGS) of sputum was performed because of her refusal of bronchoscopy (Table 2). We adjusted the dose of broad-spectrum antibiotics and used a high-flow nasal cannula with an oxygen flow rate of 55 L/min, an oxygen concentration of 60%, and a temperature was 31 °C. The patient’s symptoms of chest tightness and shortness of breath slightly improved. However, there was a significant increase in pleural fluid on the right side (Fig. 2. E1, E2). Following thoracic puncture and drainage, cytological analysis of the pleura fluid revealed malignant tumour cells. Furthermore, the pleural fluid was centrifuged and precipitated, slices were generated, basic pathology and immunohistochemistry were performed, and finally, adenocarcinoma was confirmed. Immunohistochemistry results were as follows: AE1/AE3 (+), CK7 (+), CDX-2 (+), CK20 (+), villin (+), TTF-1 (+), NapsinA (+), Calretinin (-), WT-1 (-), D2-40 (-), PGM-1 (-), Desmin (-), PAX-8 (-), GATA-3 (-), P40 (-), ALK-1A4 (-), and PD-L1 TPS = 1% (Fig. 3). The final pathological diagnosis was stage IV lung adenocarcinoma of the right lower lung with T2N2M1a classification and a performance status (PS) score of 2.

**Table 1:** Results for serum tumour marker and immune antibody levels

Tumour markers	Result	Normal range	Immune antibodies	Result	Normal range
CEA (ng/mL)	7.88	< 5	RF	< 10Ku/L	0-20IU/ml
NSE (ng/ml)	27.50	15.7–17.0	anti-Ku antibody	(-)	
ProGRP (pg/mL)	115.8	0–46	anti-PM-SCL100 antibody	(-)	
CA211 (ng/mL)	4.97	0-3.3	anti-PM-SCL75 antibody	(-)	
SCC-Ag (ng/mL)	1.00	0-1.5	anti-Jo-1 antibody	(-)	
CA125 (U/mL)	104.1	0–35	anti-SRP antibody	(-)	
CA724 (U/mL)	4.51	0 ∼ 6.7	anti-PL-7 antibody	(-)	
CA199 (U/mL)	658.8	0–37	anti-PL-12 antibody	(-)	
CA242 (U/mL)	61.1	0–20	anti-EJ antibody	(-)	
AFP (ng/mL)	3.67	< 25	anti-Ro-52 antibody	(+)	
			anti-CCP antibody	(-)	
			anti-SSA antibody	(±)	
			anti-SCL-70 antibody	(-)	
			anti-SSB antibody	(-)	
			anti-Mi-2 antibody	(-)	
			anti-dsDNA	24.3IU/mL	0-10IU/ml
			C-ANCA	(-)	
			P-ANCA	(-)	
			anti-RNP	(-)	

alpha fetoprotein, AFP; anti-neutrophil cytoplasmic antibody, ANCA; anti-double stranded DNA antibody immunoglobulin G, dsDNA IgG; carcinoembryonic antigen, CEA; carbohydrate antigen 125, CA125; carbohydrate antigen199, CA199; carbohydrate antigen724, CA724; carbohydrate antigen242, CA242; cytokeratin 19 fragment, CA211; neuron-specific enolase, NSE; pro-gastrin-releasing peptide, ProGRP; rheumatoid factor, RF; squamous cell carcinoma antigen, SCCA

**Table 2:** Next-generation sequencing results

Specimen	Microbial classification	pathogen	Number of sequences	Relative abundance
Sputum	bacteria	*Enterococcus faecium*	13	0.0
		*Klebsiella pneumoniae*	30	0.0%
		*Staphylococcus aureus*	20	0.0%
	fungus	*Candida albicans*	3077	85.5%
		*Cryptococcus neoformans*	66	1.4%
		*Candida glabrata*	30	1.4%
	virus	Human herpesvirus type 4 (EBV)	4680	92.2%
		Human herpes virus type 7	23	0.5%
		Human herpesvirus type 5 (CMV)	27	0.4%
Hydrothorax	bacteria	*Morganella morganii*	12	0.2%

## Discussion

The primary treatment for LD-CTD similar to ILD focuses on alleviating hypoxemia and symptoms to delay the decline in respiratory function. Regular high-resolution computed tomography (HRCT) screening is a minimally invasive method for detecting early LC in LD-CTD patients. HRCT has been used to identify the following CT features: peripheral bronchiectasis, mediastinal lymphadenopathy, multiple ground-glass nodules (GGNs), interlobular septal thickening, reticular abnormalities and honeycomb lungs primarily in the subpleural and basal regions [1]. This patient presented with exacerbated interstitial changes in both lower lungs following SARS-CoV-2 and influenza A virus infection, which can often be misdiagnosed or overlooked in clinical practice. Table 3 shows the differential diagnosis between ILD and viral pneumonia. In addition to cough and dyspnea, fever and nasal or systemic symptoms can be observed in patients with viral pneumonia. The imaging characteristics revealed ground glass patches in the early stage and mechanization developing to ILD in the later stage. On the other hand, when the combination of antibiotics, antiviral agents and corticosteroids fails, it is crucial to consider the possibility of pulmonary cancerous lymphangitis (PLC). Symptoms of congestive heart failure, pulmonary emboli, asthma, LC, metastasis, and radiation-induced lung disease were noted. Due to the patient’s weakness and severe condition, we were unable to perform enhanced abdominal CT and magnetic resonance imaging (MRI). Abdominal, breast, and uterine bedside ultrasound of this patient excluded metastatic cancer. PLC often has imaging characteristics similar to those of ILD and needs to be differentiated (Table 3). PLC presents with thickened interlobular septa that form polygonal arcades, accompanied by GGNs. It is characterized by the preservation of both the general and lobular architecture of the lung. The interlobular septa and peribronchovascular interstitium may exhibit uniform thickening in the early stage or nodular thickening in the later stage [9]. Multiple ground glass shadows, reticular abnormalities and cord-like structures were observed in both lungs at initial staging, which is consistent with the CT findings of LD-CTD. However, signs of peribronchovascular thickening and mediastinal adenopathy associated with pleural effusions also indicate PLC [10]. The mean survival time of patients with PLC after exhibiting pulmonary symptoms was 129 days [11]. However, the median survival time for the total CTD-ILD population exceeds 1.59 years [12], and no difference is found in the survival between patients with LD-CTD and CTD [13].

**Table 3:** Differences in clinical manifestations, radiological characteristics, disease course, treatment and prognosis among patients with ILD, PLC and viral pneumonia

Differences	ILD	PLC	Viral pneumonia
Clinical	Cough, dyspnoea, and worsening hypoxemia and exercise tolerance [14]	Similar with ILD; Tumour-related symptoms [11]	Similar with ILD; fever, fatigue, myalgia, sore throat, nasal symptoms and headache [15]
Radiological	Peripheral traction bronchiectasis, mediastinal lymphadenopathy, multiple GGNs, interlobular septal thickening, reticular abnormalities or honeycomb lungs; primarily in the subpleural and basal regions [1]	Interlobular septa thickening (early stage) and nodular thickening (later stage), preservation of lobular architecture; observed reticular, nodular, or reticulonodular patterns with coarse bronchovascular features, as well as hilar/mediastinal adenopathy associated with pleural effusions [9]	Subpleural bronchovascular bundle thickening, multifocal lung consolidation, bronchial air sign, bronchiectasis, paving stone sign and pleural thickening; observed cord, grid, patchy, nodular, even consolidation, and accompanied by GGNs, pleural effusion and mediastinal lymph adenopathy [16]
Course	Median survival was 36.2–57.7 months with anti-fibrotics [17]	Mean survival was 129days after pulmonary symptoms [11]	Mean hospitalization duration was 17.2–25.9 days [18]
Treatment	Anti-fibrotic therapy	Antitumour therapy	Anti-viral and support therapy
Prognosis	Chronic respiratory failure, lung cancer [17]	Developed fast, approximately 50% patients die within two months of their first respiratory symptoms and three weeks from admission to hospital [11]	Most cured; the mortality rates are 3 − 6.5%, H5N1 (42%) and H7N9 (30%) [15]

ground-glass nodules, GGNs; interstitial lung disease, ILD; pulmonary cancerous lymphangitis, PLC

The patient experienced a decrease in activity tolerance after developing LD-CTD in the last three years. After infection with SARS-CoV-2 and influenza A, her fibrosis was aggravated, and pleural effusion appeared. Studies have demonstrated that lung dysbiosis, such as bacterial and viral infections in the respiratory tract (including Epstein–Barr virus, cytomegalovirus, and human herpesvirus), may trigger the onset and progression of ILD [19, 20]. Patients with preexisting ILD were most likely to suffer from infection pneumonia after SARS-CoV-2 infection, and male sex and corticosteroid use were risk factors for pneumonia after infection [21]. Moreover, influenza was associated with an increased risk of lung cancer and worsened lung tumour progression [22, 23]. ILD prevalence is higher in autoimmune disease patients with anti-Ro52/SSA positivity than patients with anti-Ro52/SSA negative [24]. The demographic risk factors for the development of ILD include older age, male sex, late onset of CTD and long duration of CTD [3]. The risk factors for developing LC in patients with ILD were cigarette smoking and environmental risk factors associated with domestic and occupational exposure, and cigarette smoking concomitant with emphysema might predispose ILD patients to lung cancer (LC) [25]. Patients with CTDs are at increased risk for LC (SIR 1.27 (95% CI 0.82–1.90)) [26], and the risk of LC increases over time in rheumatoid arthritis patients [27]. The median duration from the diagnosis of CTDs to the diagnosis of LC was 17 years [28]. A history of CTD-ILD combined with heavy smoking, emphysema and nonuse of immunosuppressive therapy may be risk factors for LC, and LC development could be a poor prognostic factor in CTD-ILD patients [7, 8]. So far, there have been few clinical studies about the correlation between LD-CTD and LC.

The patient complained of joint pain and was clinically diagnosed with RA decades ago, she did not undergo any biopsy because of the stable disease and limited condition. And she did not receive any anti-inflammatory medicine until twenty-five months ago. However, we found that both RF and anti-CCP antibodies were negative, without significantly extrathoracic symptoms related to autoimmune disease (other than limited joint pain) after the patient’s final visit, but positive results were obtained for anti-SSA, anti-Ro-52 and anti-dsDNA antibodies, and initial treatments with nintedanib and glucocorticoids were effective. The symptoms did not meet strict criteria of a certain CTD according to the classification systems. Herein, LD-CTD with specific auto-antibodies but lacking adequate extrathoracic features should be considerated [29, 30]. The imaging characteristics tend to the possibility of combining non-specific interstitial pneumonia with organizing pneumonia in the later stage, but the poor responsiveness of glucocorticoid may indicate that it was not an acute exacerbation of LD-CTD, and required caution against tumors. Unfortunately, due to the rapid worsening of the disease, the patient did not receive lung biopsy and bronchoscopy. Pathology of the pleura fluid revealed positive expression of E-cadherin and MUC5AC, both of which are known to be associated with epithelial mesenchymal transition (EMT), which plays crucial roles in fibrosis and tumour progression and contributes to some extent to the fibrogenic process stemming from autoimmune diseases [31, 32].

## Conclusion

Close monitoring of serum tumour markers and screening via chest CT are crucial for patients with ILD, especially those with recurrent viral infections. Once the patient experiences worsening symptoms and signs of progressive ILD during drug administration, vigilance towards potential coexisting tumours should be maintained, and if necessary, PET-CT bronchoalveolar lavage for cytology or pathological biopsy should be performed.

## Data Availability

Data is provided within the manuscript.
